# Moving to a “flat with referee” in older age: an embodied and social transition

**DOI:** 10.1007/s10212-025-00941-x

**Published:** 2025-01-29

**Authors:** Fabienne Gfeller, Tania Zittoun

**Affiliations:** Institute of Psychology and Education, Faculté des lettres et sciences humaines, University of Neuchatel, Espace Tilo-Frey 1, 2000 Neuchatel, Switzerland

**Keywords:** Ageing, Housing for elderly people, Rupture, Transition, Embodiment, Social relations

## Abstract

Moving in older age is a critical experience in the person’s life trajectory as it may require an important reorganization of their relation to the social and material environment. In order to better understand this experience, we propose to address it drawing on the concepts of rupture and transition as developed in the frame of sociocultural lifecourse psychology. We complete this theoretical framework with the distinction between frame and space and with literature on bodies and embodiment. We present a case study conducted in a building of so-called flats with referee, a type of flats developed in the frame of a political reform addressing demographic ageing, in a Swiss canton. We focus in particular on interviews with inhabitants before and after they moved to these flats. In the analysis, we discuss two aspects of this rupture/transition which, we argue, play an important role in the persons’ experiences: firstly, the embodied dimension of the experience of rupture, which is notably related to the experience of a new physical environment; secondly, the social relations in these buildings designed especially to favor relationships among neighbors. Through this analysis, we aim at contributing to the understanding of development in older age from a sociocultural psychological perspective and to the literature on ruptures and transitions as we highlight theoretical and methodological implications.

Moving in older age is a critical experience in the person’s life trajectory as it may require an important psychological reorganization, notably related to the reconfiguration of their social and material environment. Although moving home usually involves losses, it may also represent an opportunity to move toward a place that is more adapted to the person’s social and material (notably architectural) needs. In order to better understand how people live this change, we propose to draw on a sociocultural approach to psychological development and analyze this change drawing on the concepts of rupture and transition, which notably leads us to identify learning processes. We present a case study conducted in a building of so-called flats with referee (in French, “appartements avec encadrement”)^1^ and focus in particular on interviews with inhabitants (aged between 68 and 93) before and after they moved to these flats. We discuss two aspects of this transition which, we argue, play an important role in the persons’ experiences: firstly, the embodied dimension of the experiences of rupture and transition, which is notably related to the experience of a new physical environment; secondly, the social relations which develop in these buildings designed especially to favor neighborhood relationships; as we will show, those two aspects are partially related.

## Ageing and living place

As shown by several authors, place is an important component in life trajectories in older age. While different research traditions propose a wide range of conceptualizations of places or environments and their relations with ageing, the fact that where people age and how they age are tidily interrelated is widely recognized in the literature (Martin et al., 2010; Wahl & Oswald, 2016). On the one hand, authors from different disciplines explore the meaning and the importance of home for older people:

Research on home has found home to be a reflection of a person’s ideas, a place for continuity, a place that facilitates relationships with family and friends, a centre for activities, a refuge from the outside world, an indicator of personal status, a structure, a cultural ideal and a source of gender oppression (…). To add to these multiple meanings of home, it has been argued that home takes on new meaning as one ages. (Barry et al., 2018, p. 340)

On the other hand, the importance of place in ageing is not limited to home; a larger spatial scale, such as the neighborhood, is relevant to older people’s life. Indeed, studies notably show that the configuration of places can help to maintain social life (Alidoust et al., 2019), or that care practices take place at the intersection of private and public spaces in different neighborhoods (Gabauer et al., 2022a, b). Nevertheless, the borders between home and its surroundings are sometimes porous and blurred, as illustrated by studies highlighting the importance of windows and balconies for older people who spend an increasing amount of time at home (Lenggenhager, 2017) or the importance of transitory zones and thresholds (Gabauer et al., 2022a, b).

A major trend over the last years, both in research and in policies, is an “ageing in place” discourse, which is related both to discourses on the importance of maintaining independence in old age and to the hope of limiting financial costs related to demographic ageing, and in particular costs associated with full-time institutional places of living (Ågotnes et al., 2022). However, different risks and limits regarding this trend have been identified. Firstly, ageing in place gives rise to various interpretations, which entails a risk of confusion and disappointment (Forsyth & Molinsky, 2021). Secondly, ageing in place understood as staying in one’s housing is not always optimal since there might be, for a given person, a more adapted place, where activities and care can be facilitated. Golant (2015) proposed the notion of “ageing in the right place” as an alternative to the widely used “ageing in place,” so as to reopen the possibility of moving. Thirdly, ageing in place understood as an alternative to institutionalized forms of living requires, among other, professional, financial, organizational, or caritative means that might be underestimated (Gleeson & Kearns, 2001). Finally, a reductive conception of the notion of place might neglect the social links that also constitute it:

We argue that within [the Ageing in place (AIP)] discourse, ‘place’ is predominantly interpreted as physical infrastructure, resting on a notion of the private home as ideal. (...) The relevance and need for community in a broad sense, and a community of peers in particular, is absent in such policy discourses. Perhaps as a reaction to this policy silence, a need to move from ‘ageing in place’ to ‘ageing in community’ has been voiced (Blanchard 2013), in which, for instance, older adults can gather in community dwellings. (Ågotnes et al., 2022, p. 41)

In summary, understanding older people’s environment requires considering the material dimensions of living at different scales (the house, the neighborhood, etc.), but also, and importantly, the social relationships which take place in a given space, which certain authors discuss in terms of strong and weak ties (Alidoust et al., 2019), or of community (Ågotnes et al., 2022). In addition, some authors emphasize the affective dimension of place, theorized for instance via the concept of place attachment (Hrast et al., 2020), as well as the political and economic dimensions at stake (Schwiter et al., 2018). The literature on care notably highlighted the manifold dimensions of the person’s environment in terms of social and affective interdependencies (Gabauer et al., 2022a, b), integrating spatial (Milligan & Wiles, 2010) and temporal (Antonucci et al., 2011) dimensions. In that respect, the field of study called “environmental gerontology” opened interesting avenues during the second half of the twentieth century (Moore, 2014; Schwarz, 2012; Wahl & Oswald, 2016; Wahl et al., 2003), for instance theorizing older people’s development in terms of person–environment fit. In some ways, as sociocultural psychologists, we try to renew this promising approach.

### Moving in older age

If the environment is an important component of older people’s life, it is easily understandable that moving to a new place, whether a full-time care institution or a new flat, constitutes a critical experience. Most older people are reluctant to move and perceive this event mainly as a loss of home and of social ties (Ossokina & Arentze, 2022); indeed, depending on its distance, the move implies a more or less important reorganization of the social network as well as losses (Badawy et al., 2019). As Rautenberg highlights, moving is experienced for most people as an experience of rupture:

Moving, it’s also leaving a familiar place for a foreign home. (…) It’s a slow movement of rupture and of reappropriation, in which objects, memories and habits are sorted and chosen, found and lost. If the departure is often hasty, settling in is hesitant, suspended to the experimentation of new routes and gestures that need to be acquired. (Rautenberg, 1989, p. 54, our translation)

In older age, relocating often means moving to a smaller place, which implies getting rid of many objects, and leaving a place toward which one has developed an attachment (Thalineau & Nowik, 2010). Importantly, the feeling of being at home in one’s housing is at stake as it needs to be reconstructed in the new place (Zittoun et al., 2021; Froggatt, 2001; Mallon, 2003; Salamin, 2015).

However, moving may also become an opportunity, since it might involve a change to a place that is more adapted to the person’s current needs. Motivations to willingly move in later life notably include living closer to their relatives, feeling less isolated and more secure thanks to an adapted flat, or being relieved from too heavy chores when choosing a flat with services (Thalineau, 2016); it can also be related to the need to be closer to a place or landscape where the person grew up. Hence, a move might be motivated by the need to restore, secure, or even generate capabilities, defined as “relationally shaped freedoms or opportunities that depend in complex ways on interactions between individuals and the circumstances in which they live” (Gopinath et al., 2021, p. 133), as when, for instance, people move closer to services in order to continue to do their shopping themselves (Thalineau & Nowik, 2010). A quantitative study conducted in the USA showed that as people tend to move closer to their family members, relocating in later life is in average correlated with a small increase in the person’s core social network (Badawy et al., 2019). In summary, how a move is experienced and what are its outcomes depend on many factors such as health, distance between the new and previous place (Badawy et al., 2019), reasons for the move, architectural characteristics of the previous and new place (Gopinath et al., 2021), or financial possibilities (Thalineau & Nowik, 2010). It therefore appears that moving is a critical experience in people’s trajectory, implying on the one hand parting from a place to which they are attached (and often from objects symbolically invested) as well as from persons and a social network, and on the other hand, elaborating new habits and an affective relationship to the new place. However, it also constitutes an opportunity of strengthening certain social ties, of meeting new people, and of accessing housing that is more adapted to evolving needs.

## Theoretical framework

In what follows, we draw on sociocultural psychology to address moving house in older age. This theoretical perspective allows us to examine this life period in terms of relationships between a person and their environment, focusing on the person’s unique perspective and experience. Adopting a life course perspective, we refer to the concepts of rupture and transition (Zittoun, 2006, 2012) and to the literature on frames and spaces. We complete this theoretical framework with interdisciplinary literature on bodies and embodiment.

### Ruptures and transitions

Following Rautenberg (1989), we consider moving as a potential rupture in the person’s life trajectory. From a sociocultural perspective, ruptures can be defined as events perceived as disrupting the taken-for-granted of one’s daily experiences, in one or many domains. In psychology, similar events have been coined as crises or loss of equilibrium (Erikson, 1959; Piaget, 2000). The notion of rupture encompasses a wide range of phenomena, the actual “causes” of the disruption being due to changes in the environment, in the person, or in their relationships; ruptures can also be caused by a single event or an accumulation of these (Hviid & Villadsen, 2015; Wagoner et al., 2011). Ruptures may trigger processes of transition, which designate dynamics of reorganization, possibly leading to a new state of (relative) equilibrium with the environment. Empirically, we identified three types of interrelated transformation processes implied in transitions: learning, identity transformation and repositioning, and sense-making. Studies on transitions have also examined the role of diverse resources that people use to support these dynamics, including institutional support, social relationships, personal experience, and a wide range of material and symbolic resources (Zittoun, 2006, 2008, 2022; Zittoun et al., 2012).

Moving places, whether through geographical mobility or house relocation, has been shown to often imply experiences of ruptures and trigger dynamics of transitions. In addition, this line of studies has especially shown the importance of using material objects and symbolic resources to go through the experience of moving and the ways in which such geographical moves constitute occasions of learning and development (Adams & Fleer, 2017; Langinier & Gyger Gaspoz, 2015; Levitan, 2019; Märtsin, 2019; Zittoun & Gillespie, 2015). Moreover, depending on people’s previous experiences of relocation, moving may, or not, imply a subjective experience of rupture (Levitan, 2019). However, as we show below, moving to a new flat in older age often entails a gap between people’s taken-for-granted habits and their (changing) environment that characterizes a rupture. Interestingly, the intensity of the experience of this rupture is highly variable and depends on a range of phenomena. Moreover, the subjective experience of rupture may not always correspond exactly to the moment of the physical move to the new flat.

### Frame and space

Whereas literature on ruptures and transitions gives a central importance to social relationships, the case study presented below led us to question the context in which those social relationships take place. We do so by drawing on the distinction between *frame* and *space*. The *frame* refers to the limits and boarders of a situation and evokes the rules that regulate it (Grossen & Perret-Clermont, 1992) but also designates the signs that make a situation recognizable (Grossen & Salazar Orvig, 2011). The *space* refers to the content, the multiplicity of interpretations and relations that develop inside but also outside of the frame (Grossen & Perret-Clermont, 1992). This, to some extent, meets the distinction made by some authors between house as a physical environment and home as a place experienced as supporting security and identity (Lawrence, 1987; Levitan, 2019; Sims et al., 2009). In this line, we consider, on the one hand, the building of flats with referee as a frame that includes the material constitution of the building and its formal institutional conditions, such as the regulations established at different levels; on the other hand, we consider the collective and individual ways in which the building and the flats are inhabited and experienced by their inhabitants as space. The (feeling of) home might result from the way frame and space interact and are invested through dynamics of dwelling which includes existential value (Cassin, 2015; Mallon, 2003), affects, narrative, and habits. It is also the practices developed in the formal frame that produce a socially experienced space, implying a locally shared understanding of what those mean and how to inhabit them, notably in terms of neighborhood relationships, and uses in and of the common spaces in the building. Consequently, the move to a new frame, especially if it is supposed to provide one’s new living space, is likely to be experienced as a rupture, thus triggering transitions (Zittoun & Perret-Clermont, 2009)—including learning the contours of the new frame and to orient oneself within the actual space, together with the social rules organizing it.

### Bodies

Literature on embodiment helps us to describe aspects of ruptures and transitions that are usually not taken into account in analyses of life trajectories. Embodiment is recognized as a central, primary dimension of human life and experience (Benson, 2001; Johnson, 2007), that is often neglected by psychological research, in particular when it relies on verbal methods (however, it can be apprehended discursively; see Gubrium & Holstein, 1999; Radtke et al., 2016; Stam et al., 1998). Moreover, the body is considered to be particularly salient when ageing (van Rhyn et al., 2020), especially when it becomes more fragile, prone to decline and pain. While this second aspect has a statistical reality, it does not do justice to the body as a locus of a large variety of experiences that do not necessarily depend on its performativity or validity, including experiences of joy and pleasure, nor does it make visible the varieties of ways people handle this frailty, decline, and pain, through sense-making, learning, and diverse adaptive strategies (but see Lieblich, 2014). Moreover, the representation of “the ageing body as unbounded, leaky, fragmented and lacking control” (Sandberg, 2013) has been criticized as conveying ageist and masculinist perspectives that are notably present in discourses of successful or active ageing (Bülow & Holm, 2016; Gergen & Gergen, 2010; Sandberg, 2013; Stenner et al., 2011).

Literature on body and embodiment contains a wide range of distinctions, inviting us to consider it as multidimensional or, according to Mol, as multiple (Blackman, 2021; Mol, 2002). More specifically, “the body […] is both the source and experience of subjectivity and is also the object seen, stylized, and acted upon from without” (Stam, 1998, p. 6). Therefore, in the experiences of rupture and transition, we pay a particular attention to the body as a source and experience of subjectivity, also called the body “known from within” by Harré (1994, p. 14). This raises a methodological challenge, as “[i]f the body is the very grounds of any sort of knowledge, of experience itself, (…) then the very idea that embodiment can be the clear object of a given methodology is problematic” (Brown et al., 2011, p. 494). However, this implicit, pre-reflexive body becomes objectified in certain conditions, in particular, when getting injured or exhausted, “the body [becomes] an explicit obstacle for the constant flow of interaction with the environment and lifeworld” (Tewes & Stanghellini, 2020, p. 1). We assume that similarly, in ruptures, the body can become the locus of an experience of an interruption or at least of a disturbance in the flow of interaction with the environment which leads it, to some extent, to be objectified, that is, to become an object of reflection and of activity. Moreover, in line with our sociocultural framework, we consider that this body known from within is simultaneously a social, cultural, aesthetic, emotional, historical, and political body (Blackman, 2021; Bülow & Holm, 2016; Neilson, 2012). Here again, it is rather likely that moving to a new house, which requires new embodied experiences—sensorial, mobile, and emotional—requires new learnings.

In summary, we propose to investigate learning and development in older age by examining ruptures and transitions when moving to a new home, considered in its physical, symbolic, and social dimensions, with a particular focus, first, on the embodied experience of rupture and, second, on the ruptures of the social frames and spaces in which older persons live.

## HomAge project and flats with referee

The HomAge project is a 5-year study examining older people’s development in a specific environment, namely a Swiss canton. It pays particular attention to people’s housing modes and to their formal and informal networks of care. As part of this project, we constructed a case study focused on a building of so-called flats with referee (in French, “appartements avec encadrement”). This type of flats has been developed by the canton as part of its new medico-social planning, a reform aiming at adapting cantonal health, housing, and social policies to the challenges related to demographic ageing. Drawing on a survey aimed at identifying the needs and wishes of the population of the region regarding eldercare (Barbey et al., 2009), and in line with a general trend (Milligan, 2009), the main goal of this reform was to support “ageing in place” by reducing full-time institutionalization of older persons, encouraging the construction of houses adapted to their needs, and developing and coordinating the network of institutional and caritative care (Gfeller et al., 2021; Gfeller & Zittoun, 2023). In this respect, we became interested in knowing how older persons experienced moving to a flat with referee and what “ageing in place” meant for them. Therefore, we conducted a case study in one of these new buildings, apprehending the move at the level of the ruptures people were likely to experience.

### Flats with referee

Flats with referee are an intermediary housing as defined by Thalineau (2016): it aims at being a nice place where the feeling of being at home can develop, which should facilitate the access to services and social life is fostered through the existence of a collectivity. Flats with referee were developed by the canton in response both to the identified lack of flats accessible to people with reduced mobility and to a confusing situation in which such flats were designated by several terms (“adapted,” “secured,” “protected,” “for older people”) without regulation. The construction and management of the buildings are in the hands of a variety of actors (private entrepreneurs, foundations, pension funds, etc.). Nevertheless, the legal frame constrains each commune to ensure the construction of a certain number of these flats. A label was created by the canton, which can be attributed to any place of living (flat or house) that fulfills certain criteria, based on accessibility (in terms of mobility) and on the prevention of social isolation. These criteria include the building’s accessibility for people with reduced mobility; the existence of a common room; the presence of a referee several days a week; regular, individual visits to the tenants by the referee (weekly or every fortnight); and the organization of collective activities.

While these criteria are non-negotiable conditions for obtaining the label, owners and communes are also actively encouraged (and in some cases, these might be conditions for obtaining the building permit) to construct flats that are in proximity to services (post office, shops, public transport, etc.), that are financially affordable, and following universal design guidelines going beyond accessibility for people with reduced mobility (e.g., design adapted to people with vision and hearing disorders).

There is no formal obligation for the tenants neither to accept the referee’s visits nor to participate in collective activities. However, the rent paid for the flat automatically includes a sum covering the referee’s salary. While these flats are primarily designed for older people, no formal criteria of entrance in these flats are formulated at the cantonal level, and the flats might also welcome people who have not reached the age of retirement yet, in particular people with reduced mobility.

### A case study

As part of the HomAge project, the first author conducted fieldwork for 2 years focusing on one building of flats with referee. The building has been constructed, and is owned, by a non-profit foundation and is located in a small village on a lakeside. It contains 34 apartments. Each flat has a main room with an open kitchen, one or two bedrooms, a bathroom, and a balcony or terrace. Each tenant also has access to a cellar. In order to foster social life in the building, the foundation also planned large corridors, two common terraces, and a room for craft activities. Two referees provide a 2.5-day weekly presence.

Adopting a longitudinal qualitative approach (Hermanowicz, 2016; Hollstein, 2021), this case study started approximately 1 year before the end of the construction of the building and continued for 1 year after the tenants moved in. The first author conducted interviews with tenants before and after the move, at their places. She met some of the participants up to four times, in order to follow the evolution of their situation over time. Other participants were met only once, so as to have an overview of all the inhabitants in the building without generating too much data. All the tenants were informed about the study and contacted for participation. Altogether she met 44 inhabitants living in 29 flats (some of them are couples) and conducted 61 interviews. Participants in the case study were between 55 and 93 years old. The four people aged less than 65 were not included in the analysis presented in this paper so as to focus on the population that reached the official age of retirement (in Switzerland); thus, the youngest participant included here is 68. She also conducted interviews with the referees and their supervisor (a member of the foundation), with the president of the foundation, as well as with two members of the committee. She carried out observations, in particular, in the meetings which brought together future tenants and members of the foundation, in organized collective activities, such as a concert or a meal, and in informal collective moments in the common room and on the terrace (15 observations for an approximative total of 33 h). Finally, she also collected documents such as announcements of activities in the building, pictures of the building, and leaflets for the tenants. In parallel, in order to situate the selected building in its larger context, she visited six other buildings of flats with referee. Together with other members of the HomAge project, we also conducted 16 interviews with the members of public administration promoting and labelling the flats, professional health caretakers, referees and groups of tenants from other buildings, persons interested in flats with referee but who decided not to move (yet), managers and owners of buildings, and a construction contractor.

Our research followed an abductive procedure (Reichertz, 2014) implying several movements between data production and analysis, literature review, and theoretical framework (Valsiner, 2014). We started the analysis with a transversal and collaborative analysis (Cornish et al., 2013) of the data, through which we constructed a global understanding of the flats and the diverse issues they raise. This enabled us to identify the move as a critical aspect of the tenants’ experience of the flats and thus drew on the literature on ruptures and transitions to refine our understanding. The first author conducted a thematic analysis (Braun & Clarke, 2006) on the data to identify the different aspects of the move as rupture/transition, which further led to the identification of embodiment, frame, and space as interesting concepts to continue the analysis. For the purpose of this paper, we selected particularly illustrative extracts of data.

## Moving to a flat with referee as a rupture/transition

In a sociopolitical environment in which the policy fosters “ageing in place,” we started by wondering in what respect moving to a flat with referee is experienced as rupture, thus likely to engage a transition. Unsurprisingly, all the participants experienced a rupture associated with the move to a new place. However, the affects associated with these changes, the degree to which participants felt shaken up, the ways in which the move affected their habits, and the exact moment when a rupture was experienced were highly varied, depending on numerous criteria such as the attachment to the previous place, the feeling of having a choice, and the family support. All these elements are in line with the literature on moving in later life presented above and show the need to have a better understanding of the persons’ experience of the move. Drawing on the theoretical framework presented above, we considered it at two levels: as embodied rupture and as a rupture in social life.

### Embodied aspects of the rupture/transition

The persons’ relationship to their home is also an embodied experience, and the embodied aspect of this relationship is likely to be even more salient in older age, where obstacles in the lived environment may be more strongly experienced if the body becomes frailer (van Rhyn et al., 2020). More specifically, the participants’ bodies become salient at different moments of the moving process, both during rupture experiences and processes of transition. The data extracts were selected so as to illustrate this diversity.

Firstly, before the move itself and in relation to the decision to move, the body comes to the fore when the person experiences a growing discrepancy between their body and their housing arrangements. Such discrepancy appears as relevant to many older people as they experience increasing difficulties or pain when moving around in their home, and especially when this implies taking the stairs. Many do experience a temporary or a lasting impossibility to access certain parts of their home, increased difficulties or tiredness related to the work of cleaning the house and taking care of the garden, or the fear of falling. The next extract, taken from an interview with Ms. Jungo (J) 3 months before her move, illustrates the experience of a discrepancy between the home’s characteristics and one’s body’s changing possibilities:

F (first author) Can you tell me about the advantages and obstacles at your current home, for you?

J Here, the stairs

F The stairs, that bothers you?

J Yes, the stairs, and the bathroom, because I have a bathtub. Given my back which is, I don’t tell you through which surgery I had to go, once I took a bath, and then I slipped, and then well I made such [a mess] in the bathtub. [Laughing] Because I wouldn’t leave otherwise. […] me the stairs never bothered me […] but so it is now. I can see that it gets more and more difficult.

[Ms Jungo, first interview, 3 months before the move]

Ms. Jungo’s experience of a growing discrepancy between herself and her home concerns more particularly her embodied habits in a familiar place, the change at the origin of the discrepancy being located in her body. This situation is frequent in our data. On the other hand, at times, it is the home that changes, creating such discrepancies. This occurs for instance when there is a change in the larger, yet significant, environment, such as the closure of a shop or the disappearing of an important social tie—a less frequent element in the decision to move. However, either case, people may decide to move because, somehow, the home has lost some of its taken-for-granted qualities, or some of its homeness, so that a rupture is experienced. It is as if a lived place turned back from a home to a house. In such cases, then, it seems almost natural to look for a new home, and the move appears more as a transition following the experience of rupture than a rupture by itself.

Hence, secondly, embodied experience often also becomes salient in the context of the new flat and the new building. When the inhabitants arrive in the new place, they need to get familiar with its characteristics, which notably encompasses spatial orientation, the new arrangement of furniture and objects, or acoustic features. Several tenants reported that they needed weeks or even months to get used to the new way they organized their kitchenware in the drawers and cupboards, especially when they were helped for the move and did not put the objects in the furniture themselves.

The next extract illustrates the gap between Ms. Joly’s (J) taken-for-granted spatial orientation and the organization of her new flat:

J You know it’s strange, evenings, when I watch TV, around midnight or one o’clock, suddenly I say [to myself] I should go to bed, I am LOST, but because here all is the opposite to there [in the previous flat]. There I had the back against N*[a town to the east], here I have the back towards Y* [a town to the west]. So when I stand up I don’t know anymore in which direction I am going (laughs) because yes there is some light but it is not very strong. Now I have found there are buttons I can light up the entrance hall. So I put the light on there, so I have a fixed point there. […] But I am lost, I don’t know toward which side I should turn. It will come (laughs), otherwise I will buy a compass!

F That would be a bit annoying if you have to check the compass every time

J have to find my bed (laughing). No no, it will come.

[Ms Joly, third interview, one month after the move]

We interpret the feeling of being lost as an expression of an experience of rupture; the transition then initiated involves learning to orient oneself in this new space. In this example, the experience of rupture appears after the move, as a consequence of the change of physical context. We also observe that the person could use the light of the entrance hall as a resource to support her sense of orientation and thus this learning process. Moreover, Ms. Joly also uses a more semiotic resource, humor, which enables her to take distance with this (possibly painful or scaring) experience—hence participating to sense-making.

The third extract focuses on the body circulating in and around the building, experimenting with the necessity of acquiring new habits related to its infrastructure and getting familiar with the rules and (im)possibilities of the new environment. For instance, living in the new building implies to get familiar with the door system and to learn the gestures and habits that it demands. In the building under study, if the two entrance doors can be opened from the inside, they close automatically; a key is necessary to enter back the building from the outside.

Forgetting her key led Ms. Molteni (M) to be locked out. As it happened early in the morning, she did not want to ring and to take the risk to wake up someone.

M Yesterday morning, I went down, and here it happens that I have forgotten my key. And it’s Sunday morning, early morning, 7 or around, and I couldn’t come back in anymore. (…). Nothing to do, I can’t enter the building, the [common] room [which is often open during the day and offers an alternative possibility to enter the building] is locked, nobody enters nobody goes out. I sat down, I had my rollator, […] I said too bad, I sit on it. And after some time, I had enough of sitting on this. […] I went to sit on the terrace, and I waited, and suddenly, I was almost asleep, the door of the [common] room opens. It was Ms Jacot going out to water the flowers. So I said thank you very much and I quickly went in. Quick quick!

[Ms Molteni, 4^th^ interview, one year after the move]

As this extract illustrates, forgetting one’s key has an important impact on the person’s possibilities to move and generates a series of specific physical experiences: sitting outside in the early morning on a rollator and on the terrace, starting to fall asleep, entering quickly in the building when the possibility appears.

Interestingly, 10 months earlier, Ms. Molteni spoke about her key and her need to get used to the doors:

M I also had difficulties with this, precisely the doors and all that. […], I started now these past days, […] I have to get used to it. I have to take my key with me and I have to go down and I have to go open these doors. Because I wasn’t sure to do it the right way, and then I go upstairs, there it’s the same you need the key to open the door, you always need the key. So I need to get used to handle well these doors openings. […] I’ll have to train so at it becomes easy, that it’s not difficult anymore

F you’ll get used to it

M I’ll get used to but it’s going hard, one has to go, yes yes, it’s hard but one has to go, one has to do it

[Ms Molteni, 3^rd^ interview, five weeks after the move]

In this extract, getting used to the new building is presented as a training that the person practices: taking the key when going outside and getting a firsthand experience with the doors. The body is thus perceived as a mean in the transition process; it can be trained “into” the new place, as a way to turn the new house into a home. It is presented as a difficult process, but Ms. Molteni seems confident in getting some results. Such transition thus required embodied learning, so as to be able, for example, to move around in the new place.

### Experiencing new frames and spaces: a challenge

Besides embodied experiences, the social life in the building also represents an important aspect of the inhabitants’ ruptures and transitions around the move to their new living place. Indeed, the aim of these flats, according to the canton, is in particular to induce closer social relationships among tenants than in average buildings or neighborhoods. Such aim challenges habitual ways of interacting with new neighbors and the reorganization of social networks that might be provoked by any kind of moving. Indeed, people now move to a building with a common room and regular, organized social activities, which represent a new frame. This “designed” sociality may be experienced as a rupture, as the space it opens must be collectively shaped, and simultaneously each inhabitant must appropriate it so as to feel at home, i.e., make sense of it, learn its implicit rules, and participate in shaping it.

The following extract illustrates the kind of questions that the participation in organized activities might raise (remember that there is no formal obligation to participate):

P It’s good to be here, but there is, maybe its me but we are many in this house, we meet people, err there is also many activities, so to know when one goes or doesn’t go, how is it in the others’ eyes if one doesn’t attend, me, it’s a bit me… So I have to get used to it. While in [my village] in our house we were at home, well, that’s the thing I need to get used to.

F Find your feet?

P Yes, yes, to know, that’s it, and to have the freedom to say, well here no we don’t attend, without feeling, you see

[Ms Ponti, 2^nd^ interview, two months after the move]

The frame, as shaped by the canton, the foundation, and the referees, makes participation in social activities optional. However, in a social group living in a given building, people are exposed to the gaze of others (Gillespie, 2006); dynamics of social expectations, judgement, and possibly exclusion may take place. Especially, in a new building, established rules need to be learned, appropriated, and negotiated by the inhabitants, so as to turn them into a shared culture and to organize a new sociality within collective spaces that make sense to them.

Another challenge raised by the social life in the building concerns the distribution of tasks and responsibilities in the common spaces. The following observation notes were taken during an informal meeting between three tenants having an afternoon drink in the common room, 1 year and 3 months after the opening of the building.

A person not living in the building passes through the common room to get out of the building [instead of the front door]. The inhabitants comment: why is she passing through the common room and not through the main entrance door? It is annoying because many people took this habit, and the room gets dirty. And it is ‘us’ who have to clean, not the janitor, they say; ‘we broom after activities.’ Ms Chevalley says that ‘it’s always the same ones,’ and Ms Danner adds ‘these who still can’ and that she is very glad to be able to do it, still.

This extract illustrates that the question “who does the tasks” is a topic of discussion that raises some tensions and negative affects (“always the same ones”), which also became salient at other times, for instance, when tenants complain that they do all the decoration of the common spaces, that it is a lot of work, and that other tenants criticize what they did or how they did it. Ms. Danner rather underlines that it is a luck to be still able to do it, which implicitly excuses those who do not participate in the cleaning task. In any case, at an institutional level, neither the canton nor the foundation provided guidelines that would establish an institutional frame regarding the sharing of those kinds of common tasks. As a consequence, so far, the question was never openly discussed and tasks were distributed among tenants on a voluntary and largely implicit basis. The lack of framing of the distribution of tasks by the canton and the referees offers a space of great flexibility to tenants; they can participate in activities they like (decorating) and in tasks they can perform, depending also on their investment of common spaces and activities. However, this lack of formal frame may also bring people to develop frustrations and feelings of inequity, possibly also associated with the reproduction of (gendered) social roles. The collective space then is experienced as unfair by some of the tenants. Here, perhaps, the use of collective rooms as space for addressing these issues openly may help the shaping of social life and new forms of collective learning and development (Engeström, 1999; Muller Mirza, 2009).

## Discussion

The analysis of the collected interviews and observations allows us to point out two main results that generate challenges in the implementation of the buildings of flats with referee. Both refer to tensions that we identified on the basis of our dialogical stance (Marková, 2016; Muller Mirza & Dos Santos Mamed, 2021) which assumes that differences of perspectives are inherent to any situations in which different people (and institutions) are involved, that these differences might create tensions which can be occasions for learning if they become an object of discussion and reflection (Gfeller et al., 2023; Gfeller & Zittoun, 2023).

Firstly, our research shows that moving to a flat with referees implies in general a rather important experience of rupture in the person’s life trajectory, be it before the actual physical move or as a consequence of it. Thus, most participants do not experience the move as a way to “age in place,” which, to them, means continuing to live in the place where they have lived for many years, which they know well and to which they are attached. At the cantonal level, however, the flats are conceived as a way of fostering “ageing in place,” understood as an alternative to full-time institutionalization. While the flats indeed represent such an alternative for some inhabitants, most of them experience the move as an important rupture during which the sense of home, and thus of possibly “ageing in place,” is put at stake in a quite radical way. Our research thus shows a discrepancy between the perspective of the designers and promotors of flats with referee (in particular, the cantonal institutions) and the perspectives of (future) inhabitants of these flats. The diversity of meanings attached to the notion of ageing in place is an issue already highlighted by Forsyth and Molinsky (2021). Reading this discrepancy through the concepts of rupture and transitions allows us to highlight specific consequences of this misunderstanding that constitute a challenge for the development of this type of flats. Indeed, for all the participants, the move constitutes a transition, and describing the flats as a possibility to “age in place” tends to overshadow the important work done to go through this transition.

Secondly, our research also shows some of the transition processes related both to the material and spatial features of the buildings of flats with referee, experienced bodily, and to the social life within those buildings. On the one hand, living in a new frame and its space affords new embodied experiences and requires new activities—orienting oneself or learning to maneuver the entrance door. The transition to a new house thus requires a period of try-and-fail—getting lost in one’s flat, being locked out—and the identification of material and semiotic resources to develop the adequate strategies and new and situated skills. This of course also requires sense-making and identity changes, at times dealt with humor.

On the other hand, these buildings designed to foster a more vivid social life than in individual houses or average buildings create new situations for the participants. The canton asks referees to support the creation of social life, and the collective spaces are also meant to foster shared activities. Although participation in collective activities is formally not compulsory, this does not elude reciprocal expectations between tenants, who might expect others to participate, since this is a building where social life was advertised. Referees might also have difficulties to find a balance between encouraging inhabitants to participate so as to foster their social life, but also respecting their choice not to participate. Moreover, the definition and repartition of work in the common room are not formally defined by the cantonal frame and must therefore be organized by the inhabitants. Thus, in the frame established by the canton, the space can be invested in different ways (Zittoun et al., 2013). In consequence, a collective culture regarding participation needs to be elaborated. Also, people have to learn, or to re-learn, to live with a social group on a daily basis—something many of them have not done for a very long time.

The new houses, as social frames and spaces, experienced by embodied persons, engaged de facto in a form of social and collective life, thus require both individual learning and collective knowledge building—learning the rules and building the community (Engeström, 1999; Heath, 2004).

## Conclusion

Drawing on a sociocultural approach to the life course, and in particular on the concepts of rupture and transition, we analyzed older persons’ moves to a building of flats with referee. We highlighted that while the policy makers in charge of developing a new medico-social planification consider these flats as a form of ageing in place, older people moving to such a flat experienced this move as a rupture. We highlighted the importance of the embodied and social dimensions of rupture and transitions. Such findings have both theoretical and methodological implications.

At a theoretical level, our paper contributes to the understanding of learning in older age. Research on ruptures and transitions has mainly focused on identity, learning, and meaning-making as symbolic/semiotic processes that play a major role in these periods of the life course (Zittoun, 2006, 2008; Zittoun et al., 2012). Our study highlights that, at least in some cases, it is worth paying attention to embodied experiences and to materiality, as bodies may be the locus of a felt discrepancy between the person and their environment that is characteristic of a rupture. Moving is a change in one’s life in which materiality is central (Gyger Gaspoz, 2014; Levitan, 2019). When studying ageing population, embodied ruptures may be particularly important, and the body and its changes tend to increasingly become an object of thought, while it mostly maintains a pre-reflexive status in the life of younger adults (van Rhyn et al., 2020). In that case, learning and sense-making primarily require reflecting on one’s embodied activities in space and one’s gestures when dealing with its material features; interestingly, people may be quite creative in finding the resources to support these learning and developmental processes. Altogether, this calls for a better inclusion of materiality, spatiality, and the body in the developmental psychology of older adults.

On a methodological level, our findings call for a more nuanced identification of rupture and transitions in our fieldworks. In our data, in some cases, the rupture that triggers the move to a new place is the growing mismatch between the body’s possibilities and the place of living; in other cases, the experience of an embodied rupture is the consequence of the arrival in the new place of living. Experienced ruptures can thus be the cause, or the consequence of a geographical movement (Cangià, 2018, 2021; Wyss et al., 2023; Author 2 & Gillespie, 2015). Such temporal gaps between a visible change (the move) and the subjectively experienced rupture have also been identified in the case of changes of foodways around vegetarianism (Author 1, 2020). This implies that processes of transition might start (in our study, several months) before the visible change (in our study, the move). Such observations also call to pay specific attention to the actual disruptive experiences. In short, our observation calls for more careful attention to the potential discrepancy between actual geographical moves and psychological experiences of ruptures.

## Data Availability

Data are not accessible for the sake of personal data protection.

## Footnotes

1 The term “referee” was chosen in relation to the name given to the professionals working in those flats, in French, les référentes, rather than in relation with “encadrement.” Although this translation might be questionable, we decided to keep it for two reasons. Firstly, this person has, in our interpretation, a role that is somehow close to the one of a sport referee, or more broadly “a person or organization that helps to find a fair answer to a disagreement” (Oxford dictionary) in their role of management of collective life; and of “a person to whom reference is made, esp. for an opinion, information, or a decision” (Collins English Dictionary) in their task of personal guidance of the inhabitants. Secondly, we want to keep coherence with other publications on the same project.
